# Supplementary material for: Natural sequence variants of yeast environmental sensors confer cell-to-cell expression variability
Source: Mol Syst Biol. 2013 Oct 8;9:695. doi: 10.1038/msb.2013.53 (PMC3817403; doi:10.1038/msb.2013.53)
Supplement: Supplementary Information [file msb201353-s1.pdf]

Supplementary Information

Natural sequence variants of yeast environmental sensors confer cell-to-cell expression variability

Steffen Fehrmann, Hélène Bottin-Duplus, Andri Leonidou, Esther Mollereau, Audrey Barthelaix, Wu Wei, Lars M. Steinmetz and Gaël Yvert.

TABLE OF CONTENT

Supplementary Table 1 …………………….……….... 2

Supplementary Table 2 …………………….……….... 3

Supplementary Table 3 …………………….……….... 5

Supplementary Figure 1……………………….…….... 7

Supplementary Figure 2…………………….……….... 8

Supplementary Table 1. Genotypes of allele-replacement strains for *ERC1*

		SNP	SNP	SNP	SNP	SNP	SNP	INDEL	SNP	SNP
**distance from ATG**		219	294	580	750	777	822	1628	1669	1798
**BYallele**		G	G	T	T	A	A	A	C	T
**RMallele**		A	T	G	C	C	C	-	T	C
**GY1019**	*RM-ERC1BY*	A	T	G	C	C	C	A	C	T
**GY1020**	*RM-ERC1BY*	G	G	T	T	A	A	A	C	T
**GY1023**	*BY-ERC1RM*	A	T	G	C	C	C	-	T	C
**GY1024**	*BY-ERC1RM*	G	G	G	C	C	C	-	T	T

Genomic DNA was amplified using primers 1I37 and 1H90 and sequenced.
Only polymorphisms present in the targeting construct are represented.

Supplementary Table 2: Strains used in this study

Name	General Background	Specific Genotypes	Source
BY4712	S288c	*MATa leu2Δ0*	*(Brachmann et al, 1998)*
BY4713	S288c	*MATalpha leu2Δ0*	*(Brachmann et al, 1998)*
BY4716	S288c	*MATalpha lys2Δ0*	*(Brachmann et al, 1998)*
BY4719	S288c	*MATa trp1Δ63 ura3Δ0*	*(Brachmann et al, 1998)*
PHO84L259P	S288c	*MATalpha leu2Δ0 ura3Δ0 PHO84-L259P*	*(Perlstein et al, 2007)*
FYC2-9C	S288c	*MATalpha his3Δ200*	*B. Dujon*
GY51	S288c	*MATalpha lys2Δ0 HIS3:(natMX + Pmet17-yEGFP3)*	*(Ansel et al, 2008)*
GY53	RM11-1a	*MATa leu2Δ0 ura3Δ0 amn1Δ::HYG ho::KAN HIS3:(natMX +* P_{met17}*-yEGFP3):HIS3*	*(Ansel et al, 2008)*
GY159	S288c (94%), RM11-1a (6%)	*MATa leu2Δ0 lys2Δ202 trp1Δ63 ura3Δ0 HIS3:(natMX +* P_{met17}*-yEGFP3):HIS3*	*(Ansel et al, 2008)*
GY172	S288c	*MATa trp1Δ63 HIS3:(natMX +* P_{met17}*-yEGFP3):HIS3*	*(Ansel et al, 2008)*
GY174	S288c (94%), RM11-1a (6%)	*MATa ura3Δ0 HIS3:(natMX +* P_{met17}*-yEGFP3):HIS3*	*(Ansel et al, 2008)*
GY246	S288c	*MATa leu2Δ0 ura3Δ0 HIS3:(natMX +* P_{met17}*-yEGFP3):HIS3*	*(Ansel et al, 2008)*
GY321	S288c	*MATa lys2-128d dst1Δ::hisG HIS3:(natMX +* P_{met17}*-yEGFP3):HIS3*	*(Ansel et al, 2008)*
GY512	S288c	*MATa his3Δ200 lys2-128d dst1Δ::hisG*	GY321 x FYC2-9C
GY601	RM11-1a	*MATa leu2Δ0 URA3 amn1Δ::HYG ho::KAN HIS3:(natMX +* P_{met17}*-yEGFP3):HIS3*	*(Ansel et al, 2008)*
GY768 (X7)	S288c (99%), RM11-1a (1%)	*MATalpha lys2Δ0 HIS3:(natMX +* P_{met17}*-yEGFP3):HIS3*	*This study*
GY769 (Z7)	S288c (99%), RM11-1a (1%)	*MATalpha leu2Δ0 HIS3:(natMX +* P_{met17}*-yEGFP3):HIS3*	*This study*
GY793 (W7)	S288c (99%), RM11-1a (1%)	*MATalpha lys2Δ0 HIS3:(natMX +* P_{met17}*-yEGFP3):HIS3*	*This study*
GY911	S288c (99.5%), RM11-1a (0.5%)	*MATa trp1Δ63 HIS3:(natMX +* P_{met17}*-yEGFP3):HIS3 ePTL1-RM ePTL7-RM*	GY768 x GY51
GY915	S288c except one locus	*MATalpha lys2Δ0 trp1Δ63 HIS3:(natMX +* P_{met17}*-yEGFP3):HIS3 ePTL13-RM*	GY793 x GY172
GY919	S288c except one locus	*MATa trp1Δ63 HIS3:(natMX +* P_{met17}*-yEGFP3):HIS3 ePTL8-RM*	GY769 x GY172
GY921	S288c except two loci	*MATa leu2Δ0 HIS3:(natMX +* P_{met17}*-yEGFP3):HIS3 ePTL8-RM chrXV-563573-703057-RM*	GY769 x GY172
GY926	S288c except one locus	*MATa lys2Δ0 trp1Δ63 HIS3:(natMX +* P_{met17}*-yEGFP3):HIS3 ePTL1-RM*	GY911 x GY51
GY927	S288c except one locus	*MATa LYS2. trp1Δ63 HIS3:(natMX +* P_{met17}*-yEGFP3):HIS3 ePTL7-RM*	GY911 x GY51
GY929	S288c	*MATalpha leu2Δ0 thr1Δ::kanMX4 HIS3:(natMX +* P_{met17}*-yEGFP3):HIS3*	Euroscarf Y16608 X GY172
GY930	S288c except one locus	*MATa trp1Δ63 ePTL8-RM*	GY919 x BY4716
GY931	S288c except one locus	*MATa trp1Δ63 cox6Δ::kanMX4 ePTL8-RM*	This study
GY935	S288c except one locus	*MATa lys2Δ0 trp1Δ63 HIS3:(natMX +* P_{met17}*-yEGFP3):HIS3 ePTL7-RM*	GY911 x GY51
GY937	S288c except three loci	*MATalpha leu2Δ0 trp1Δ63 HIS3:(natMX +* P_{met17}*-yEGFP3):HIS3 ePTL8-RM ePTL13-RM chrXV-563573-703057-RM*	GY921 x GY915
GY939	S288c except one locus	*MATa/MATalpha leu2Δ0/LEU2 trp1Δ63/TRP1 THR1/thr1Δ::kanMX4 HIS3/HIS3:(natMX +* P_{met17}*-yEGFP3):HIS3 cox6Δ::kanMX4/COX6 ePTL8-RM/ePTL8-BY*	GY931 X GY929
GY943	S288c except four loci	*MATalpha leu2Δ0 trp1Δ63 HIS3:(natMX +* P_{met17}*-yEGFP3):HIS3 ePTL7-RM ePTL8-RM ePTL13-RM chrXV-563573-703057-RM*	GY937 x GY927
GY975	S288c	*MATalpha leu2Δ0 hoΔ::(*P_{act1}*-GFP+KanMX4)*	*This study*
GY1002	S288c	*MATalpha lys2Δ0 HIS3:(NatMX4 +*P_{mup1RM}*-yEGFP3):HIS3*	*This study*
GY1003	S288c	*MATalpha lys2Δ0 HIS3:(NatMX4 +*P_{mup1RM}*-yEGFP3):HIS3*	*This study*
GY1004	S288c	*MATalpha lys2Δ0 HIS3:(NatMX4 +*P_{mup1RM}*-yEGFP3):HIS3*	*This study*
GY1005	S288c	*MATalpha lys2Δ0 HIS3:(NatMX4 +*P_{mup1BY}*-yEGFP3):HIS3*	*This study*

GY1006	S288c	*MATalpha lys2Δ0 HIS3:(NatMX4 +P_{mup1BY}-yEGFP3):HIS3*	*This study*
GY1007	S288c	*MATalpha lys2Δ0 HIS3:(NatMX4 +P_{mup1BY}-yEGFP3):HIS3*	*This study*
GY1019	RM11-1a	*MATa leu2Δ0 ura3Δ0 amn1::HYG ho::KanMX HIS3:(natMX + P_{met17}-yEGFP3):HIS3 ERC1-BY*[(i)]	*This study*
GY1020	RM11-1a	*MATa leu2Δ0 ura3Δ0 amn1::HYG ho::KanMX HIS3:(natMX + P_{met17}-yEGFP3):HIS3 ERC1-BY*[(i)]	*This study*
GY1023	S288c	*MATa leu2Δ0 ura3Δ0 HIS3:(natMX + P_{met17}-yEGFP3):HIS3 ERC1-RM*[(i)]	*This study*
GY1024	S288c	*MATa leu2Δ0 ura3Δ0 HIS3:(natMX + P_{met17}-yEGFP3):HIS3 ERC1-RM*[(i)]	*This study*
GY1156	S288c	*MATa trp1Δ63 hoΔ::(P_{act1}-GFP+TRP1)*	*This study*
GY1205	RM11-1a	*MATa leu2Δ0 ura3Δ0 amn1::HYG ho::KanMX HIS3:(natMX + P_{met17}-yEGFP3):HIS3 MUP1-BY*	*This study*
GY1206	RM11-1a	*MATa leu2Δ0 ura3Δ0 amn1::HYG ho::KanMX HIS3:(natMX + P_{met17}-yEGFP3):HIS3 MUP1-BY*	*This study*
GY1207	RM11-1a	*MATa leu2Δ0 ura3Δ0 amn1::HYG ho::KanMX HIS3:(natMX + P_{met17}-yEGFP3):HIS3 MUP1-BY*	*This study*
GY1208	RM11-1a	*MATa leu2Δ0 ura3Δ0 amn1::HYG ho::KanMX HIS3:(natMX + P_{met17}-yEGFP3):HIS3 MUP1-BY*	*This study*
GY1284	S288c except one locus	*MATalpha lys2Δ0 ePTL13-RM*	GY915 x BY4712
GY1296	S288c	*MATalpha lys2Δ0 HIS3:(natMX + P_{met17}-yEGFP3):HIS3 pho84Δ::KanMX4*	Euroscarf Y16524 x GY172
GY1302	S288c except one locus	*MATalpha lys2Δ0 ePTL13-RM-atr1Δ::KanMX4*	*This study*
GY1303	S288c except one locus	*MATalpha lys2Δ0 ePTL13-RM-pho84Δ::KanMX4*	*This study*
GY1306	S288c	*MAT? HIS3:(natMX + P_{met17}-yEGFP3):HIS3 PHO84-L259P*	PHO84L259P x GY172
GY1307	S288c	*MAT? HIS3:(natMX + P_{met17}-yEGFP3):HIS3 PHO84-L259P*	PHO84L259P x GY172
GY1308	S288c	*MAT? HIS3:(natMX + P_{met17}-yEGFP3):HIS3 PHO84-L259P*	PHO84L259P x GY172
GY1309	S288c except one locus	*MATalpha trp1Δ63 HIS3:(natMX + P_{met17}-yEGFP3):HIS3 ePTL13-RM-atr1Δ::KanMX4*	GY1302 x GY172
GY1310	S288c except one locus	*MATalpha lys2Δ0 HIS3:(natMX + P_{met17}-yEGFP3):HIS3 ePTL13-RM-pho84Δ::KanMX4*	GY1303 x GY172
GY1325	S288c	*MATalpha lys2Δ0 HIS3:(NatMX4 +P_{atr1BY}-yEGFP3):HIS3*	*This study*
GY1326	S288c	*MATalpha lys2Δ0 HIS3:(NatMX4 +P_{atr1BY}-yEGFP3):HIS3*	*This study*
GY1327	S288c	*MATalpha lys2Δ0 HIS3:(NatMX4 +P_{atr1RM}-yEGFP3):HIS3*	*This study*
GY1328	S288c	*MATalpha lys2Δ0 HIS3:(NatMX4 +P_{atr1RM}-yEGFP3):HIS3*	*This study*
GY1330	S288c	*MATa leu2Δ0 ura3Δ0 HIS3:(natMX +Pmet17-yEGFP3):HIS3 ATR1-RM*	*This study*

(i): See Supplementary Table 1 for the detailed *ERC1* genotype of strains GY1019, GY1020, GY1023 and GY1024.

REFERENCES:

Ansel J, Bottin H, Rodriguez-Beltran C, Damon C, Nagarajan M, Fehrmann S, Francois J, Yvert G (2008) Cell-to-cell stochastic variation in gene expression is a complex genetic trait. *PLoS Genet* **4:** e1000049

Brachmann CB, Davies A, Cost GJ, Caputo E, Li J, Hieter P, Boeke JD (1998) Designer deletion strains derived from Saccharomyces cerevisiae S288C: a useful set of strains and plasmids for PCR-mediated gene disruption and other applications. *Yeast* **14:** 115-132

Perlstein EO, Ruderfer DM, Roberts DC, Schreiber SL, Kruglyak L (2007) Genetic basis of individual differences in the response to small-molecule drugs in yeast. *Nat Genet* **39:** 496-502

Supplementary Table 3: Primers used in this study.

ID	Sequence (5' to 3')	Comments
1G84	GCACGGCGCCAACTGAAAAG	RFLP marker of PTL chrVII position 599377 (SfcI)
1G85	GGCCTGCCTCAAAATCGGAG	RFLP marker of PTL chrVII position 599377 (SfcI)
1G88	ATGGCATCGTCTAGTGATTA	RFLP marker of PTL chrI position 47045 (TfiI)
1G89	CTGAGAGACTCTCTTCCCTT	RFLP marker of PTL chrI position 47045 (TfiI)
1G90	AAAGACATCCGGATAGCAAA	RFLP marker of candidate locus chrV position 203237 (HphI)
1G91	TTTGCATCTGCAGTGACAAC	RFLP marker of candidate locus chrV position 203237 (HphI)
1G92	TTACTCCAAAAGGCTCCTGA	RFLP marker of candidate locus chrVI position 263987 (MfeI)
1G93	CGATAACTCGGTACGATCAA	RFLP marker of candidate locus chrVI position 263987 (MfeI)
1G96	CACCACCAAACAGTCCTGGA	RFLP marker of candidate locus chrIX position 199365 (TfiI)
1G97	CCGGATTCCAGTGATGCTTA	RFLP marker of candidate locus chrIX position 199365 (TfiI)
1H01	CTATAGTTCATATACCATAGGC	RFLP marker of candidate locus chrXIII position 807167 (BbsI)
1H02	GTATGCAACTGCTATCATTC	RFLP marker of candidate locus chrXIII position 807167 (BbsI)
1H03	TACCGATGGTTAAAAATGA	Marker of PTL chrXV position 625582 (HRM)
1H04	GGGGATTATGTATTTGAAAC	Marker of PTL chrXV position 625582 (HRM)
1H23	CATAGCCAAGGCCAGTTGATTGA	RFLP marker of PTL chrVIII position 160869 (BsaJI)
1H24	ACCGGCACCAATCATGGATGA	RFLP marker of PTL chrVIII position 160869 (BsaJI)
1H27	TGCACTGTGGCTTAGCGGAAA	RFLP marker of PTL chrXIII position 40087 (MseI)
1H28	GAACCGAGCGATGACGATTTGA	RFLP marker of PTL chrXIII position 40087 (MseI)
1H89	TCGCTATACGACAGCTGCATGA	
1H90	ATGCCACATGGCGAAACCCTTA	
1I12	tctttcattatgtgagagtttaaaaaccagaaactacatcatcgaaaaag-GCTTTTCACGCCTGCAGACTACAT	
1I13	cgcccaatacgcaaaccgcctctccccgcgcgttggccgattcattaatg-TACGTCATTCGACCCTGAAGGAGT	
1I29	cgcccaatacgcaaaccgcctctccccgcgcgttggccgattcattaatg-tctaga-AGGGCAACCCAACCGGTAATAGAA	
1I32	tctttcattatgtgagagtttaaaaaccagaaactacatcatcgaaaaag-TCACTTGGTCGCCAACTTCAAGG	
1I35	taattcaaccaaaattgggacaacaccagtgaataattcttcacctttta-GACAT-TGCTCCGCCTTTGTTTTC	
1I36	ccgcgttctaacgacaatatgtccatatggtgcactctcagtacaatctgc-CTCCACAAATGCCCCCACAAAC	
1I37	CCTTCCTATACCGGCAATGACGAT	
1I80	GGAAAAGTTGGGTTGGAAGGATGA	
1I81	GGTTGGCGTAATTCTCCTGGGAAA	
1I98	tcaaccaaaattgggacaacaccagtgaataattcttcacctttaGACAT-TATTATTACCTGTTTGAGATTCTC	

1I99	ccgcgttctaacgacaatatgtccatatggtgcactctcagtacaatctgc<br>-CGTCATTAAGACAAGCTCC	
1J06	CCGAGCGATCCTGAATACGCTTTA	
1J07	TGGGGAGGGCAAGAAATTGGTT	
1J08	ACTACCCGGAGATGACTTCAAACG	
1J09	GACAATTGAGGCGCAGGATAAACG	
1J10	tctttcattatgtgagagtttaaaaaccagaaactacatcatcgaaaaag-<br>AGCTCCATCCGCTGTACAGTTCTA	
1J11	cgcccaatacgcaaaccgcctctccccgcgcgttggccgattcattaat<br>g-GGAAAAGATGCCATCAGCCATGAC	
1J12	TCATGGCCCAATACCTTGCACA	RFLP marker of ATR1 (ApoI)
1J13	CCTATCACGCCCAAAACAGAACCA	RFLP marker of ATR1 (ApoI)
1J15	TCTTATCTTGGTTGCTGCTT	To genotype PHO84-L259P polymorphism
1J16	CTTTTCGCCATCTTGTTCTTG	To genotype PHO84-L259P polymorphism

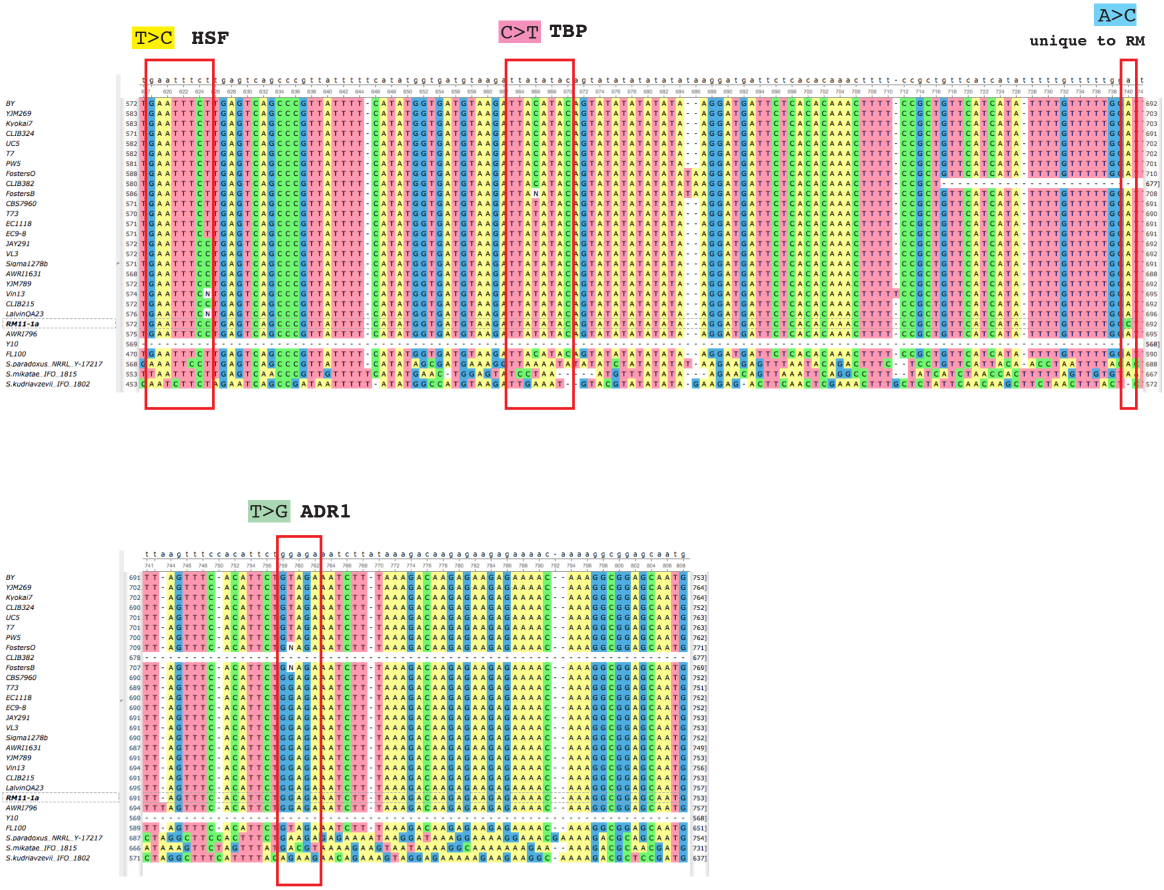

Supplementary Figure 1. Sequence alignment of the promoter region of MUP1.

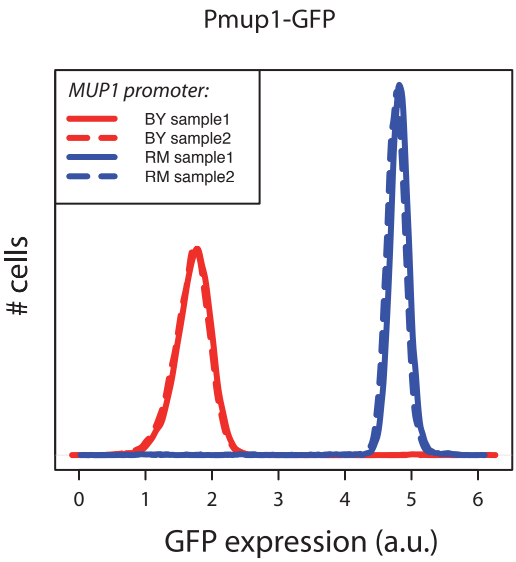

Supplementary Figure 2.
Representative distributions of GFP expression levels obtained by flow cytometry on populations of cells harboring a Pmup1-GFP integrated reporter. Two strains are shown, one carrying the BY variant of the MUP1 promoter (GY1006, red) and one carrying the RM variant (GY1002, blue). Each strain was cultured in duplicates (plain and dashed line).
